# Main Effects of Diagnoses, Brain Regions, and their Interaction Effects for Cerebral Metabolites in Bipolar and Unipolar Depressive Disorders

**DOI:** 10.1038/srep37343

**Published:** 2016-11-21

**Authors:** Hai-Zhu Tan, Hui Li, Chen-Feng Liu, Ji-Tian Guan, Xiao-Bo Guo, Can-Hong Wen, Shao-Min Ou, Yin-Nan Zhang, Jie Zhang, Chong-Tao Xu, Zhi-Wei Shen, Ren-Hua Wu, Xue-Qin Wang

**Affiliations:** 1 Department of Physics and Computer Applications, Shantou University Medical College, Shanou, 515041, China; 2 Southern China Center for Statistical Science, Sun Yat-Sen University, Guangzhou, 510275, China; 3 Department of Medical Imaging, 2nd Affiliated Hospital, Shantou University Medical College, Shantou, 515041, China; 4 Mental Health Center; Shantou University Medical College, Shantou, 515000, China; 5 Department of Statistical Science, School of Mathematics, Sun Yat-Sen University, Guangzhou, 510275, China; 6 Provincial Key Laboratory of Medical Molecular Imaging, Guangdong, Shantou, 515041, China; 7 Zhongshan School of Medicine, Sun Yat-Sen University, Guangzhou, 510080, China

## Abstract

Previous studies suggested patients with bipolar depressive disorder (BDd) or unipolar depressive disorder (UDd) have cerebral metabolites abnormalities. These abnormalities may stem from multiple sub-regions of gray matter in brain regions. Thirteen BDd patients, 20 UDd patients and 20 healthy controls (HC) were enrolled to investigate these abnormalities. Absolute concentrations of 5 cerebral metabolites (glutamate-glutamine (Glx), N-acetylaspartate (NAA), choline (Cho), myo-inositol (mI), creatine (Cr), parietal cortex (PC)) were measured from 4 subregions (the medial frontal cortex (mPFC), anterior cingulate cortex (ACC), posterior cingulate cortex (PCC), and parietal cortex (PC)) of gray matter. Main and interaction effects of cerebral metabolites across subregions of gray matter were evaluated. For example, the Glx was significantly higher in BDd compared with UDd, and so on. As the interaction analyses showed, some interaction effects existed. The concentrations of BDds’ Glx, Cho, Cr in the ACC and HCs’ mI and Cr in the PC were higher than that of other interaction effects. In addition, the concentrations of BDds’ Glx and Cr in the PC and HCs’ mI in the ACC were statistically significant lower than that of other interaction effects. These findings point to region-related abnormalities of cerebral metabolites across subjects with BDd and UDd.

Bipolar depressive disorder (BDd) is an aspect of bipolar disorder (BD), which is a severe illness with unpredictable cycles of depression and manic episodes^1^,^2^,^3^. Unipolar depressive disorder (UDd), which is a major depressive disorder (MDd), is a severe illness with negative mood^3^,^4^. In clinical practice, BDd patients are often misdiagnosed as UDd because the etiology is still unclear, and we know little about the underlying pathology of BDd and UDd^3^,^4^. Hence, it calls for unbiased and reliable diagnostic methods to distinguish BDd and UDd. Previous studies have examined biochemical abnormalities in subregions of gray matter in BDd and UDd patients using proton magnetic resonance spectroscopy (^1^H-MRS)^5^,^6^.

^1^H-MRS offers a safe, noninvasive, and nonradioactive method^7^. It can help us obtain information about cerebral metabolites, including glutamate-glutamine (Glx), N-acetylaspartate (NAA), choline (Cho), creatine (Cr), and myo-inositol (mI). LCModel (version 6.3 1 A) is used to estimate and analyze metabolite concentrations from localized *in vivo* proton NMR spectra^8^. Alterations in cerebral metabolites have been demonstrated to serve as potential biomarkers that distinguish healthy, BDd, and UDd cohorts^9^. For example, Glu concentrations in the left Heschl’s Gyrus and Planum Temporale of the superior temporal gyrus of patients with schizophrenia and BDd have been proved to be lower than healthy cohorts^10^. Comparing to schizophrenia patients and healthy cohorts, lower Glu, NAA, Cr and Inositol in patients with BDd suggested that a possible location-specific abnormality was existed in the dominant hemisphere of auditory cortices in patients with BDd^11^. As the main excitatory transmitter in the human brain, altered Glx was found in gray matter areas of patients with BDd^12^,^13^. NAA, considering close associations with the energetics of mitochondria and neuronal loss, has been found decreased in patients with BDd and UDd^14^,^15^,^16^. Cho is a membrane compound and increased choline may indicate increased membrane turn-over^17^. Many neuropsychiatric diseased are related with abnormal single transduction. Cho signaling may have impact on single transduction^14^,^16^. Hence, choline may have the potential to serve as a diagnostic biomarker in adolescent BDd and UDd^17^. As a putative marker of glial cells, the level of mI has been shown to be significantly reduced in UDd while increased in BDd paitents^7^,^14^. Cr plays a key role in maintaining energy stores, and its level is considered relatively constant^14^,^16^,^18^, but recent research have report that Cr levels are not stable in some psychiatric populations^11^,^19^.

As mentioned above, cerebral metabolites are considered biomarkers for distinguishing BDd and UDd patients, and the ROIs are associated with them as well. A series of studies on the neurobiology of BDd and UDd were done within the last decades. Most of them simply focused on independently comparing cerebral metabolites among various groups in each brain region^20^,^21^,^22^. Some studies conducted an interaction analysis on between the effect of diagnosis by brain region on the Glx/Cr level or the Glu/Cr level^23^. However, to our knowledge, few studies have comprehensively examined the relationships of cerebral metabolites’ absolute concentration in the various subregions of gray matter among 3 cohorts. Based on the literature reviewed above, we hypothesized that abnormalities of metabolites in multiple sub-regions of gray matter in brain region might be happened in patients with BDd and UDd. And then the main effects of diagnoses, brain regions, and their interaction for cerebral metabolites in patients with BDd and UDd would be presented when comparing to HC.

## Patients and Methods

### Patients Selection

Our study was approved by the Shantou University psychiatric research ethics committee (see the attach file of the project approved document). All our clinical trials adhered to the protocols of Shantou University psychiatric research ethics committee. The methods applied in this study were closely performed according to the guide of the proposal of applying multiple neuroimaging technology to diagnose bipolar and unipolar patients (see the attach file of the proposal of project).

In this context, we mainly analyzed acquired data of 5 cerebral metabolites from 4 brain regions in gray matter among three independent cohorts, including 13 un-medicated patients with BDd, 20 un-medicated patients with UDd, and 20 healthy controls (HC). Patients with BDd and UDd were enrolled from the outpatient clinic at Shantou University Mental Health Center, Shantou, China, from August 2014 to July 2015. Twenty HC were recruited volunteers. Demographic and clinical characteristics of the 3 cohorts are compared in Table 1 (See Supplementary Appendix).

DSM-IV criteria, which were based on the Structured Clinical Interview for DSM-IV Patient Edition (SCID-P), were used to confirm BDd and UDd patients with a score of 17 or greater on the 17-item Hamilton Depression Rating Scale (HDRS). Written informed consent was obtained from all subjects. Patients were excluded if they declined to sign the informed consent. In addition, we excluded participants who were younger than 18 years or older than 60 years, who had a history of neurological disorders, a serious medical condition, or a ferrous implant or pace maker, and those who were pregnant or breastfeeding. All participants were right-handed.

### Cerebral Metabolites and Subregions of Gray Matter

**Figure 1:**
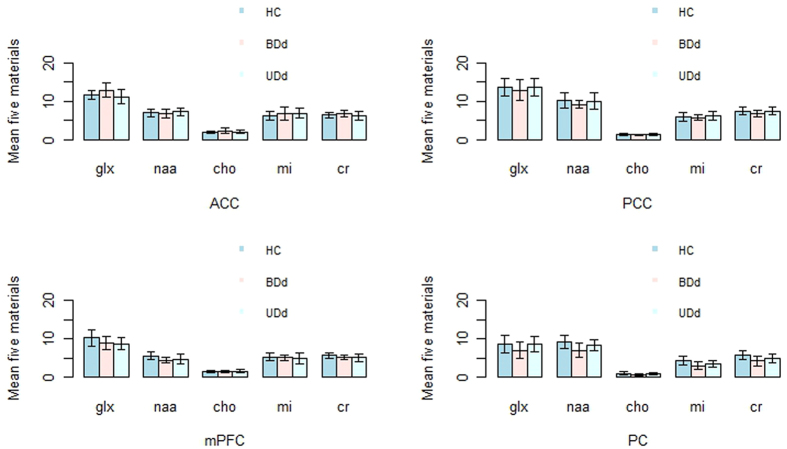
Concentrations of Metabolites in Different Brain Regions from the 3 Controls.

MRS with a 2-dimensional multi-voxel technique (2D MRSI) was performed to measure simultaneously cerebral metabolites from multi-voxels within a single slice. In this study, 4 subregions were measured in gray matter, including the medial frontal cortex (mPFC), anterior cingulate cortex (ACC), posterior cingulate cortex (PCC), and parietal cortex (PC) (see Figs 1 and 2 in Supplementary Appendix)^24^. All of the regions of interest (ROIs) were located in gray matter areas. These ROIs are essential to mood regulation and cognitive processes.

All participants underwent 2D MRSI exam of the brain as well as a structural MRI scan. A 15-mm axial slice was used to cover all of the ROIs in gray matter. ^1^H MRSI acquisitions were conducted on a 3 T GE Signa MR scanner (General Electric Medical Systems, USA) scanner equipped with a standard 8-channel head coil array, and the spectroscopic data were obtained from the slice with a nominal voxel size of 2.8 ml. Concentrations of 5 cerebral metabolites were determined in the 4 volume-localized spectrums.

### Statistical Analysis

#### Group Difference Analysis of Cerebral Metabolites **a**cross Brain Regions

We compared the 5 cerebral metabolites among 3 independent cohorts in 4 brain regions and analyzed the differences to assess their diversities. We initially checked normality and homogeneity assumptions using normality and homogeneity of variance tests, respectively (the results see Table 1). When the assumptions were satisfied, analysis of variance (ANOVA) was used to compare the differences among the 3 groups. If difference among groups existed, Tukey’s HSD test was then used to determine which groups had significant differences among the cerebral metabolites and the degree of the difference between groups was calculated. As long as a hypothesis was unsatisfied, the Kruskal-Wallis test, a nonparametric method, was used to assess the differences among groups. Wilcoxon test was preceded to identify the significant difference between two groups.

#### Interaction Effect of Diagnosis by Brain Region

The aim of our study was to find out whether an interaction of effect of each adjusted cerebral metabolites of 4 brain regions and 3 diagnoses cohort existed separately. After the normality test for 5 cerebral metabolites (the results see the Supplementary Appendix), we found that NAA and Cho were unsatisfied with the assumptions. We do Box-Cox transformation for NAA and Cho. And then a linear regression model of each of these 5 adjusted cerebral metabolites (the detailed method See Supplementary Appendix) was used to determine whether an interaction effect of each adjusted cerebral metabolite of 4 brain regions and 3 diagnosis cohorts existed. In addition, an interaction diagram plot, which clearly illustrated the combined effect of the 3 cohorts and brain regions, was drawn using the ggplot2 of R software.

## Results

### Cerebral Metabolites and Subregions of Gray Matter

Figure 1 depicts the distribution of the 5 cerebral metabolites in 4 brain regions for the 3 independent cohorts. “Mean ± SD” was used to describe variability within controls.

### **Group Differ**ence Analysis of Cerebral Metabolites in 4 Subregions

Group differences in 5 cerebral metabolites are displayed in Table 2. Multiple comparison analysis on the above selected significant cerebral metabolites was performed, and the results were shown in Table 3.

### ACC

In the 3-group difference analysis, there was no statistically significant group difference except for Glx (see Table 2). Table 3 suggests that the BDd group had higher Glx levels in the ACC when compared with the UDd group.

### PCC

According to the normality and homogeneity analysis, all cerebral metabolites except NAA satisfied the assumptions. Then, group difference analysis was performed in a manner identical to that for the ACC. Table 2 shows that there were no significant group differences for any metabolites in the PCC.

### **m**PFC

From Table 2, we found that significant group differences existed for both Glx and NAA in the mPFC. Table 3 showed that the Glx and NAA levels of the UDd group and the NAA level of the BDd group were lower than those of the HC group.

### PC

In addition to Glx, all considered metabolites had significant group differences (see Table 2). From Table 3, we found that PC levels of NAA, Cho, mI, and Cr of the BDd group, as well as the level of Cr of the UDd group were lower when compared with the healthy group.

### Interaction Effect of Diagnosis by **Subr**egion **of Gray Matter**

**Figure 2:**
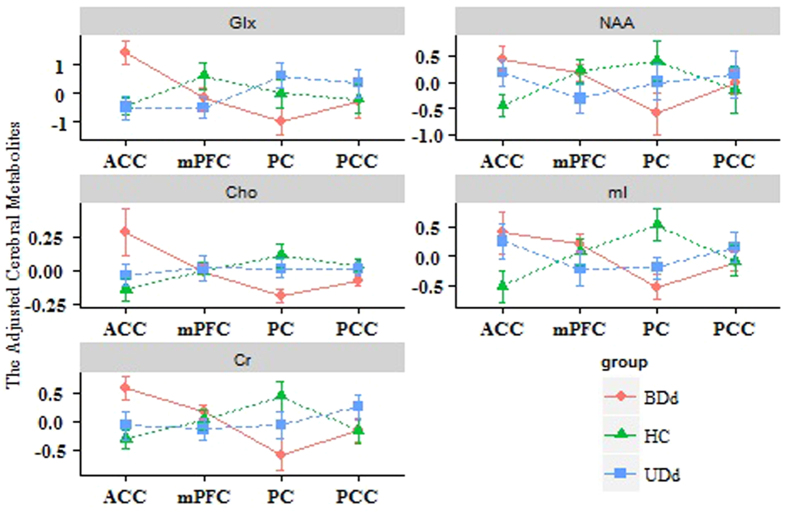
Diagnosis by brain region interaction effect of adjusted cerebral metabolites.

The interaction effect of brain regions and the 3 diagnosis groups was tested to see whether there were differences in each cerebral metabolite among specific brain regions across 3 groups. Table 4 presents the results of 3 diagnosis groups (BDd, UDd, HC), 4 brain regions, and interactions of the 5 independently adjusted cerebral metabolites. The interaction plot in Fig. 2 clearly illustrates the interaction effects.

### Glx

From results in Table 4 and Fig. 2, we found that there was an interaction effect for the cerebral metabolite, Glx. For example, the interaction effect of BDd: ACC indicated that the adjusted Glx level for BDd patients were measured more from the ACC region (estimate = 1.408) than that of other interaction effects in Table 4. It was also demonstrated by the P-value of unadjusted Glx (P-value = 0.009) in Fig. 2.

### NAA

From Table 4, Fig. 2, we found that there were interaction effects existed in the ACC and PC regions for 3 diagnosis groups. For example, the level of BDds’ NAA was measured less in PC region than that of other interaction effects.

### Cho

Interaction effects of Cho were displayed in Table 4 and Fig. 2). The interaction of BDd:ACC indicated that the adjusted Cho level for BDd patients were discovered more from the ACC region (estimate = 0.189) than that of other interaction effects. The interaction of BDd:PC indicated that the adjusted Cho level for BDd patients were less found from the PC region (estimate = −0.187) than that of other interaction effects.

### mI

In Table 4, The interaction of HC:ACC indicated that the adjusted mI level for HC were measured less from the ACC region (estimate = −0.519) than that of other interaction effects. In addition, the interaction of HC:PC suggested that the adjusted mI level for HC was found more from the PC region (estimate = 0.531) than that of other interaction effects. Figure 2 also proved that the interaction effects existed for HCs’ levels of mI in the ACC and PC regions.

### Cr

The interaction of BDd:ACC indicated that the adjusted Cr level for BDd patients were measured more from the ACC region (estimate = 0.579) than that of other interaction effects. However, the adjusted Cr level for BDd patients were less found from the PC region (estimate = −0.585). Moreover, the interaction of HC:PC indicated that the adjusted Cr level for HC were discovered from the PC region (estimate = 0.411) (see Table 4). Figure 2 also shows the interaction effects of Cr.

## Discussion

All participants are un-medicated subjects in our study. Medications have major influence on the level of brain biochemical metabolites^25^. For example lithium have been found that it has significant effects on Glu/Cr and Glx/Cr concentrations^26^,^27^ and cho membrane transport^28^. The effect of antidepressant treatment might cause NAA levels increased significantly on medial frontal cortex in MDD patients^29^. Therefore, in order to better understand the brain metabolism changes in the subregions of gray matter on patients with BDd and UDd, participants with un-medicated BDd patients and UDd patients were enrolled in this study. The methodology of using a ratio of one cerebral metabolism compared with other metabolite was applied in earlier studies, just like Cho/Cr ratios^2^,^13^,^30^, NAA/Cr ratios^2^,^6^, or Glx/Cr ratios^2^, etc However, they could not accurately reflect the change of cerebral metabolite concentrations^30^. Recent results revealed that using ratio rather than absolute concentration levels of metabolites was a technical limitation^23^. Hence, we combined the 2D-MRSI (magnetic resonance spectroscopy imaging) and LCModel software^21^,^22^ in gray matter areas to measure the absolute concentration.

Most studies focused on certain cerebral metabolite concentrations in single voxel sections (such as ACC^31^,^32^ and mPFC^32^,^33^) among groups. Researchers utilized multi-voxel technique to obtain multiple 1H-MRS spectra and evaluate multiple brain areas, just like we measured multi-voxel sections in gray matter areas. Also, we analyzed the main effect of diagnoses, brain regions for cerebral metabolites in BDd and UDd patients. Abnormalities of the 5 cerebral metabolites in different gray matter were existed in BDd and UDd patients. In the ACC region, we observed a significantly higher concentration of Glx when comparing BDd patients with UDd patients. That is, elevated glutamate in the ACC was associated with BDd^22^,^34^,^35^ and reduced Glx in the ACC was associated with patients with UDd. These findings were congruent with several previous reports^22^,^34^,^35^,^36^,^37^,^38^. These alterations of Glx suggested that a primary abnormality was presented in the ACC brain region between these two groups. In the PCC region, there were no significant group differences among 5 metabolites. The interpretation of this result is stillFZ difficult because studies on the PCC area are still few^39^,^40^. In addition, we found that the concentrations of Glx and NAA were reduced in UDd patients as well as NAA concentration was reduced in BDd patients in the mPFC region when compared to the HC cohort. This result was in accordance with prior studies demonstrating changes of NAA concentration in patients with BDd^41^,^42^. In contrast to the HC cohort, decreased NAA, Cho, mI, and Cr in patients with BDd and decreased Cr in the UDd cohort were found in the PC region in our studies. These findings are in agreement with previous studies^22^. Reductions of NAA, Cho, mI and Cr concentration in PC region may serve as some functional signals for neuronal damage or impaired function in patients with BDd. With the interaction effect analyses, Glx concentrations in patients with BDd always were measured lower in the ACC region. The changes of Glx concentrations in mPFC of patients with UDd were higher than that of other interaction effects. This result was confirmed with the result of main effects analyses. In a recent study, lower Cho/Cr was reported in the bilateral ventral prefrontal white matter of patients with MDD^43^. We found that Cho concentrations in the PC region of patients with BDd always were lower than that of other interaction effects. Our finding in turn, was a good evidence of that previous study. As the present study showed, the HCs’ mI concentrations in ACC were higher and in PC region were lower than that of other interaction effects. Some postmortem study have similar reports^44^,^45^. The Cr level for BDd patients in PC regions were lower than that of other interaction effects. It coincided with the earlier research about decreased Cr concentrations were seen in the left hippocampus of BD patients^46^,^47^. The interaction effects analyses of diagnosis by brain region for five cerebral metabolites are our preliminary research work. It is difficult for us to mine and interpret the clinical significance of differences at present. More clinical trials are needed to further validated and explained.

Some possible methodological limitations of the current study should be taken into consideration. Unable to find a good way to distinguish glutamate from glutamine, creatine and phosphocreatine signals accurately is the first limitation. The second limitation is that the sample size was relatively small in our study. An additional limitation is that the brain metabolite changes are not measured before and after treatment of BDd and UDd participants.

In summary, the findings of this study indicate abnormalities of cerebral metabolites in subregions of gray matter in UDd and, in particular, BDd. In the follow-up experiments, a larger number of subjects must be enrolled and longitudinal study of BDd and UDd participants may be introduced to see whether there are brain metabolite changes before and after treatment. In addition, this investigation will have to be validated in clinical trials.

## Additional Information

**How to cite this article**: Tan, H.-Z. *et al.* Main Effects of Diagnoses, Brain Regions, and their Interaction Effects for Cerebral Metabolites in Bipolar and Unipolar Depressive Disorders. *Sci. Rep.* **6**, 37343; doi: 10.1038/srep37343 (2016).

**Publisher’s note:** Springer Nature remains neutral with regard to jurisdictional claims in published maps and institutional affiliations.

## Supplementary Material

Supplementary Information

## Figures and Tables

**Table 1:** The P-Value of Normlity Test and Homogeneity Test.

	ACC		mPFC		PC		PCC	
	Normlity	Homogeneity	Normlity	Homogeneity	Normlity	Homogeneity	Normlity	Homogeneity
Glx	0.526	0.198	0.051	0.801	0.811	0.801	0.999	0.837
NAA	0.851	0.898	0.000	0.047	0.418	0.047	0.157	0.789
Cho	0.000	0.001	0.069	0.851	0.369	0.851	0.025	0.133
mI	0.329	0.648	0.133	0.169	0.044	0.169	0.191	0.261
Cr	0.780	0.162	0.112	0.877	0.798	0.877	0.336	0.753

**Table 2:** The Results of ANOVA Analysis.

	ACC		PCC		mPFC		PC	
	F-value	P-value	F-value	P-value	F-value	P-value	F-value	P-value
Glx	4.469	**0.016**	0.833	0.659	4.293	**0.019**	2.981	0.060
NAA	0.813	0.449	1.496	0.473^*^	5.264	**0.008**	6.033	**0.004**
Cho	2.68	0.262^*^	3.097	0.213	0.246	0.783	7.829	0.020^*^
MI	1.234	0.300	0.759	0.474	2.748	0.253*	7.879	**0.001**
Cr	1.153	0.324	1.763	0.182	2.753	0.073	7.296	**0.002**

*Kruskal-Wallis H test was used.

**Table 3:** Analysis of Multiple Comparisons Among Groups.

Metabolites	Inter-group	ACC		mPFC		PC	
		Diff	P-value	Diff	P-value	Diff	P-value
Glx	HC-BDd	—	0.109	—	0.090	—	—
	HC-UDd	—	0.561	−1.59	**0.023**	—	—
	BDd-UDd	−1.76	0.012	—	0.957	—	—
NAA	HC-BDd	—	—	−1.11	**0.015**	−2.08	**0.003**
	HC- UDd	—	—	−0.87	**0.033**	—	0.338
	BDd- UDd	—	—	—	0.820	—	0.079
Cho	HC-BDd	—	—	—	—	0.780	**0.005**
	HC- UDd	—	—	—	—	—	0.368
	BDd- UDd	—	—	—	—	—	0.992
MI	HC-BDd	—	—	—	—	−1.40	**0.001**
	HC- UDd	—	—	—	—	−0.87	**0.026**
	BDd- UDd	—	—	—	—	—	0.332
Cr	HC-BDd	—	—	—	—	−1.57	**0.001**
	HC- UDd	—	—	—	—	−0.90	**0.049**
	BDd- UDd	—	—	—	—	—	0.265

- insignificant difference, p-value > 0.05.

**Table 4:** Interaction Effect of Adjusted Cerebral Metabolites.

	Coefficient	P-value	Metabolite
BDd:ACC	1.408	0.012	Glx
BDd:ACC	0.189	0.012	Cho
BDd:PC	−0.187	0.013	Cho
HC:ACC	−0.519	0.044	mI
HC:PC	0.531	0.039	mI
BDd:ACC	0.579	0.033	Cr
HC:PC	0.441	0.043	Cr
BDd:PC	−0.585	0.031	Cr

Note: insignificant interaction effects (P-value > 0.05) were not displayed in Table 4.

## References

[b1] Zhong S. *et al.* Similarities of biochemical abnormalities between major depressive disorder and bipolar depression: a proton magnetic resonance spectroscopy study. Journal of affective disorders 168, 380–386 (2014). 25106035 10.1016/j.jad.2014.07.024

[b2] Haldane M. & Frangou S. New insights help define the pathophysiology of bipolar affective disorder: neuroimaging and neuropathology findings. Progress in Neuro-Psychopharmacology and Biological Psychiatry 28, 943–960 (2004). 15380855 10.1016/j.pnpbp.2004.05.040

[b3] Cuellar A. K., Johnson S. L. & Winters R. Distinctions between bipolar and unipolar depression. Clinical psychology review 25, 307–339 (2005). 15792852 10.1016/j.cpr.2004.12.002 PMC2850601

[b4] Forty L. *et al.* Clinical differences between bipolar and unipolar depression. The British Journal of Psychiatry 192, 388–389 (2008). 18450667 10.1192/bjp.bp.107.045294

[b5] Strakowski S. M., DelBello M. P., Adler C., Cecil K. M. & Sax K. W. Neuroimaging in bipolar disorder. Bipolar disorders 2, 148–164 (2000). 11256682 10.1034/j.1399-5618.2000.020302.x

[b6] Drevets W., Ongür D. & Price J. Neuroimaging abnormalities in the subgenual prefrontal cortex: implications for the pathophysiology of familial mood disorders. Molecular psychiatry 3, 220–226, 190–221 (1998). 9672897 10.1038/sj.mp.4000370

[b7] Castillo M., Smith J. K. & Kwock L. Correlation of myo-inositol levels and grading of cerebral astrocytomas. American Journal of Neuroradiology 21, 1645–1649 (2000). 11039343 PMC8174883

[b8] Provencher S. W. Estimation of metabolite concentrations from localized *in vivo* proton NMR spectra. Magnetic resonance in medicine 30, 672–679 (1993). 8139448 10.1002/mrm.1910300604

[b9] Taylor M. J. Could glutamate spectroscopy differentiate bipolar depression from unipolar? - Journal of Affective Disorders. Journal of Affective Disorders 167, 80–84 (2014). 25082118 10.1016/j.jad.2014.05.019

[b10] Atagun M. I. *et al.* P.3.b.014 Investigation of Heschl gyrus and planum temporale in patients with schizophrenia and bipolar disorder: a 1H MRS study. European Neuropsychopharmacology 24, S499–S499 (2014).

[b11] Atagün M. İ. *et al.* Investigation of Heschl’s gyrus and planum temporale in patients with schizophrenia and bipolar disorder: A proton magnetic resonance spectroscopy study. Schizophrenia Research 161, 202–209 (2015). 25480359 10.1016/j.schres.2014.11.012 PMC4308441

[b12] Hoekstra R. *et al.* Bipolar mania and plasma amino acids: increased levels of glycine. European neuropsychopharmacology 16, 71–77 (2006). 16023835 10.1016/j.euroneuro.2005.06.003

[b13] Xu J. *et al.* Neurochemical abnormalities in unmedicated bipolar depression and mania: A 2D 1 H MRS investigation. Psychiatry Research: Neuroimaging 213, 235–241 (2013). 23810639 10.1016/j.pscychresns.2013.02.008 PMC3729606

[b14] Winsberg M. E. *et al.* Decreased dorsolateral prefrontal N-acetyl aspartate in bipolar disorder. Biological psychiatry 47, 475–481 (2000). 10715353 10.1016/s0006-3223(99)00183-3

[b15] Chang K. *et al.* Decreased N-acetylaspartate in children with familial bipolar disorder. Biological psychiatry 53, 1059–1065 (2003). 12788251 10.1016/s0006-3223(02)01744-4

[b16] Öngür D. *et al.* Abnormal glutamatergic neurotransmission and neuronal-glial interactions in acute mania. Biological psychiatry 64, 718–726 (2008). 18602089 10.1016/j.biopsych.2008.05.014 PMC2577764

[b17] Shi X. F. *et al.* Anterior cingulate cortex choline levels in female adolescents with unipolar versus bipolar depression: a potential new tool for diagnosis. Journal of Affective Disorders 167, 25–29 (2014). 25082110 10.1016/j.jad.2014.05.051 PMC4699311

[b18] Alchanatis M. *et al.* Frontal brain lobe impairment in obstructive sleep apnoea: a proton MR spectroscopy study. European Respiratory Journal 24, 980–986 (2004). 15572542 10.1183/09031936.04.00127603

[b19] Öngür D., Prescot A. P., Jensen J. E., Cohen B. M. & Renshaw P. F. Creatine abnormalities in schizophrenia and bipolar disorder. Psychiatry Research Neuroimaging 172, 44–48 (2009). 19239984 10.1016/j.pscychresns.2008.06.002 PMC2729651

[b20] Frye M. A. *et al.* Reduced concentrations of N-acetylaspartate (NAA) and the NAA–creatine ratio in the basal ganglia in bipolar disorder: A study using 3-Tesla proton magnetic resonance spectroscopy. Psychiatry Research: Neuroimaging 154, 259–265 (2007). 17346949 10.1016/j.pscychresns.2006.11.003

[b21] Yücel M. *et al.* Anterior cingulate glutamate–glutamine levels predict symptom severity in women with obsessive–compulsive disorder. Australian and New Zealand Journal of Psychiatry 42, 467–477 (2008). 18465373 10.1080/00048670802050546

[b22] Frye M. A. *et al.* Increased anterior cingulate/medial prefrontal cortical glutamate and creatine in bipolar depression. Neuropsychopharmacology 32, 2490–2499 (2007). 17429412 10.1038/sj.npp.1301387

[b23] Xu J. *et al.* Neurochemical abnormalities in unmedicated bipolar depression and mania: A 2D 1 H MRS investigation. Psychiatry Research Neuroimaging 213, 235–241 (2011). 10.1016/j.pscychresns.2013.02.008 PMC3729606 23810639

[b24] Jitrapakdee S. & Wallace J. C. The biotin enzyme family: conserved structural motifs and domain rearrangements. Current Protein and Peptide Science 4, 217–229 (2003). 12769720 10.2174/1389203033487199

[b25] Zhong S. *et al.* Similarities of biochemical abnormalities between major depressive disorder and bipolar depression: A proton magnetic resonance spectroscopy study. Journal of Affective Disorders 168, 380–386 (2014). 25106035 10.1016/j.jad.2014.07.024

[b26] Friedman S. D. *et al.* Lithium and valproic acid treatment effects on brain chemistry in bipolar disorder. Biological Psychiatry 56, 340–348 (2004). 15336516 10.1016/j.biopsych.2004.06.012

[b27] Brennan B. P. *et al.* Rapid enhancement of glutamatergic neurotransmission in bipolar depression following treatment with riluzole. Neuropsychopharmacology Official Publication of the American College of Neuropsychopharmacology 35, 834–846 (2010). 19956089 10.1038/npp.2009.191 PMC3055603

[b28] Pleul O. & Müller-Oerlinghausen B. Lithium therapy and the turnover of phosphatidylcholine in human erythrocytes. European Journal of Clinical Pharmacology 31, 457–462 (1986). 3816926 10.1007/BF00613524

[b29] Gonul A. S. *et al.* The effect of antidepressant treatment on N -acetyl aspartate levels of medial frontal cortex in drug-free depressed patients. Progress in Neuro-Psychopharmacology and Biological Psychiatry 30, 120–125 (2006). 16236417 10.1016/j.pnpbp.2005.08.017

[b30] Wu R. H. *et al.* Brain choline concentrations may not be altered in euthymic bipolar disorder patients chronically treated with either lithium or sodium valproate. Annals of General Psychiatry 3, 13 (2004). 10.1186/1475-2832-3-13 PMC509421 15283867

[b31] Auer D. P. *et al.* Reduced glutamate in the anterior cingulate cortex in depression: an *in vivo* proton magnetic resonance spectroscopy study. Biological psychiatry 47, 305–313 (2000). 10686265 10.1016/s0006-3223(99)00159-6

[b32] Théberge J. *et al.* Glutamate and glutamine in the anterior cingulate and thalamus of medicated patients with chronic schizophrenia and healthy comparison subjects measured with 4.0-T proton MRS. American Journal of Psychiatry (2014). 10.1176/appi.ajp.160.12.2231 14638596

[b33] Rao J. S., Harry G. J., Rapoport S. I. & Kim H.-W. Increased excitotoxicity and neuroinflammatory markers in postmortem frontal cortex from bipolar disorder patients. Molecular psychiatry 15, 384–392 (2010). 19488045 10.1038/mp.2009.47 PMC2844920

[b34] Mullins P. G., Rowland L. M., Jung R. E. & Sibbitt W. L. Jr. A novel technique to study the brain’s response to pain: proton magnetic resonance spectroscopy. Neuroimage 26, 642–646 (2005). 15907322 10.1016/j.neuroimage.2005.02.001

[b35] Gussew A. *et al.* Time-resolved functional 1H MR spectroscopic detection of glutamate concentration changes in the brain during acute heat pain stimulation. Neuroimage 49, 1895–1902 (2010). 19761852 10.1016/j.neuroimage.2009.09.007

[b36] Dager S. R. *et al.* Brain metabolic alterations in medication-free patients with bipolar disorder. Archives of general psychiatry 61, 450–458, doi: 10.1001/archpsyc.61.5.450 (2004). 15123489

[b37] Eastwood S. L. & Harrison P. J. Markers of glutamate synaptic transmission and plasticity are increased in the anterior cingulate cortex in bipolar disorder. Biological psychiatry 67, 1010–1016, doi: 10.1016/j.biopsych.2009.12.004 (2010). 20079890 PMC2868790

[b38] Chen G., Henter I. D. & Manji H. K. Presynaptic glutamatergic dysfunction in bipolar disorder. Biological psychiatry 67, 1007–1009, doi: 10.1016/j.biopsych.2010.03.027 (2010). 20457307 PMC2872125

[b39] Maddock R. J. & Buonocore M. H. MR spectroscopic studies of the brain in psychiatric disorders. Curr Top Behav Neurosci 11, 199–251 (2012). 22294088 10.1007/7854_2011_197

[b40] Pomarolclotet E. *et al.* Brain functional changes across the different phases of bipolar disorder. British Journal of Psychiatry the Journal of Mental Science 206, 136–144 (2014). 10.1192/bjp.bp.114.152033 25497296

[b41] Alcauter S. Increased myo-inositol in the posterior cingulate cortex in first-episode major depressive patients. Advances in Bioscience & Biotechnology 04, 45–52 (2013).

[b42] Manji H. K., Moore G. J. & Chen G. Clinical and preclinical evidence for the neurotrophic effects of mood stabilizers: implications for the pathophysiology and treatment of manic-depressive illness. Biological psychiatry 48, 740–754 (2000). 11063971 10.1016/s0006-3223(00)00979-3

[b43] Deicken R. F., Eliaz Y., Feiwell R. & Schuff N. Increased thalamic N-acetylaspartate in male patients with familial bipolar I disorder. Psychiatry Research: Neuroimaging 106, 35–45 (2001). 11231098 10.1016/s0925-4927(00)00083-4

[b44] Zhang Y. *et al.* A MRS study of metabolic alterations in the frontal white matter of major depressive disorder patients with the treatment of SSRIs. Bmc Psychiatry 15, 1–7 (2015). 25934495 10.1186/s12888-015-0489-7 PMC4458012

[b45] Shimon H., Agam G., Belmaker R. H., Hyde T. M. & Kleinman J. E. Reduced frontal cortex inositol levels in postmortem brain of suicide victims and patients with bipolar disorder. American Journal of Psychiatry 154, 1148–1150 (1997). 9247405 10.1176/ajp.154.8.1148

[b46] Coupland N. J. *et al.* Decreased Prefrontal Myo -Inositol in Major Depressive Disorder. Biological Psychiatry 57, 1526–1534 (2005). 15953489 10.1016/j.biopsych.2005.02.027

[b47] Haarman B. C. M. *et al.* Volume, Metabolites and Neuroinflammation of the Hippocampus in Bipolar Disorder - A combined Magnetic Resonance Imaging and Positron Emission Tomography Study. Brain Behavior & Immunity 56, 21–33 (2015). 10.1016/j.bbi.2015.09.004 26348581

[b48] Frey B. N. *et al.* Corrected values of brain metabolites for the article: ‘Abnormal cellular energy and phospholipid metabolism in the left dorsolateral prefrontal cortex of medication-free individuals with bipolar disorder: an *in vivo* 1H MRS study’. Bipolar Disorders 9, 119–127(119) (2007). 17543030 10.1111/j.1399-5618.2007.00454.x

