# Supplementary material for: Main Effects of Diagnoses, Brain Regions, and their Interaction Effects for Cerebral Metabolites in Bipolar and Unipolar Depressive Disorders
Source: Sci Rep. 2016 Nov 21;6:37343. doi: 10.1038/srep37343 (PMC5116758; doi:10.1038/srep37343)
Supplement: Supplementary Information [file srep37343-s1.pdf]

Main Effects of Diagnoses, Brain Regions, and their Interaction Effects for Cerebral Metabolites in Bipolar Depressive Disorders

Hai-Zhu Tan [1,3,8], Hui Li [5,6,8], Chen-Feng Liu[2,3], Ji-Tian Guan[5], Xiao-Bo Guo[2,3],Can-Hong Wen[2,3], Shao-Min Ou[1], Yin-Nan Zhang[6], Jie Zhang[6], Chong-Tao Xu[6], Zhi-Wei Shen[5], Ren-Hua Wu[5,7,*],Xue-Qin Wang [2,3,4,*]

[1] Department of Physics and Computer Applications, Shantou University Medical College，Shanou, 515041, China;

[2] Department of Statistical Science, School of Mathematics, Sun Yat-Sen University, Guangzhou, 510275, China;

[3] Southern China Center for Statistical Science, Sun Yat-Sen University, Guangzhou, 510275, China;

[4] Zhongshan School of Medicine, Sun Yat-Sen University, Guangzhou, 510080, China;

[5] Department of Medical Imaging, 2nd Affiliated Hospital, Shantou University Medical College, Shantou, 515041,China.

[6] Mental Health Center; Shantou University Medical College, Shantou, 515000, China.

[7] Provincial Key Laboratory of Medical Molecular Imaging, Guangdong, Shantou, 515041, China.

* To whom correspondence should be addressed: wangxq88@mail.sysu.edu.cn or cjr.wurenhua@vip.163.com

[8] These authors contributed equally to this work.

Supplementary Appendix

Contents

Figure

Fig.1. Representative placement of the MRSI slice on a T2-weighted sagittal images and typical voxel selection for the region of interest analysis is illustrated on the same subjects' T1 fluid attenuated inversion recovery (FLAIR) image covering ACC, mPFC, PC, and PCC.

Fig.2. Representative 1H-MRS spectra from the four brain regions of mPFC (A), ACC (B), PCC (C), and PC(D) and the four brain regions were shown in axial T1 FLAIR image in the left column of the figure.

Table

Table.1. Clinical Characteristics of Participants in the 3 Cohorts

Methods

A linear regression model of each of the 5 adjusted cerebral metabolites

Figure

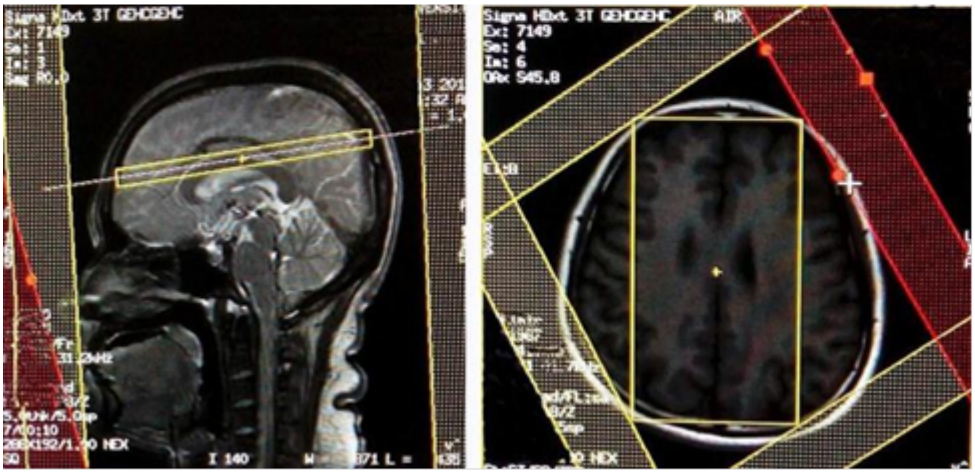

Fig.1. Representative placement of the MRSI slice on a T2-weighted sagittal images (left). Typical voxel selection for the region of interest analyses is illustrated on the same subjects' T1 fluid attenuated inversion recovery (FLAIR) image covering ACC, mPFC, PC, and PCC(right).

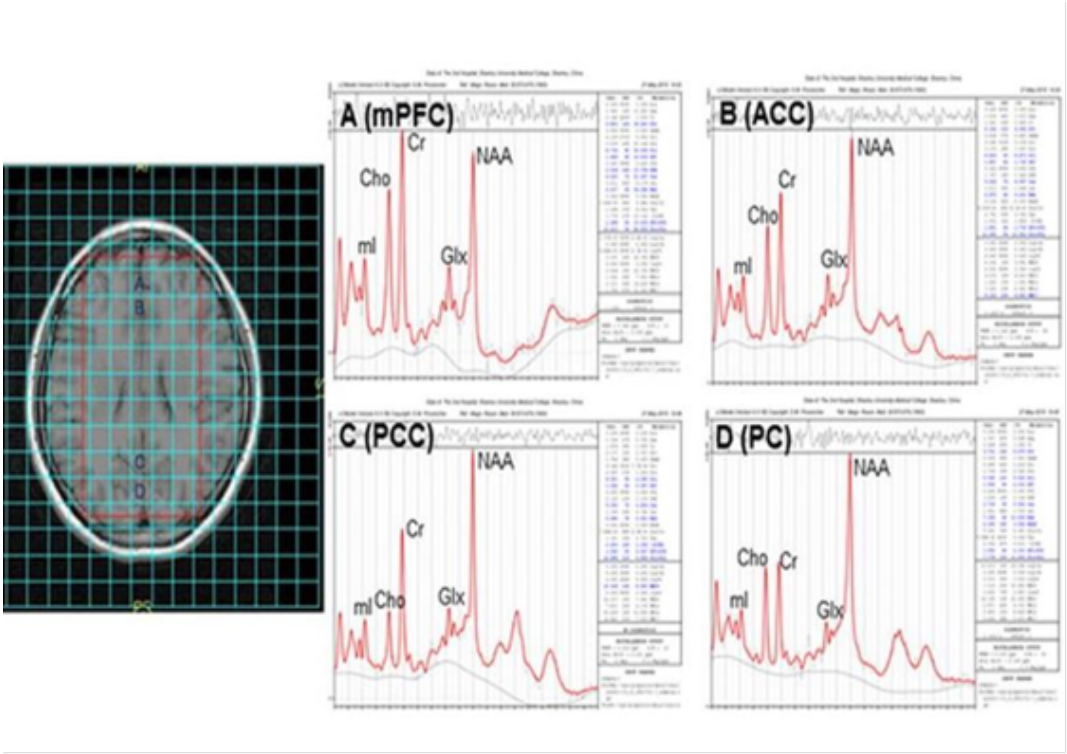

Fig.2. Representative 1H-MRS spectra from the four brain regions of mPFC (A), ACC (B), PCC(C), and PC(D). The four brain regions were shown in axial T1 FLAIR image in the left column of the figure.

Table

Table 1 Clinical Characteristics of Participants in the 3 Cohorts

	BDd Control (n = 13)		UDd Control (n = 20)		Healthy Control (n = 20)	
	mean	sd	mean	sd	mean	sd
Age(year)	31	7.6	27.95	9.06	31.65	11.4
Education(year)	11.69	3.99	10.5	3.52	13.1	3.89
Ld* (month)	108	54.99	56.4	29.42		
Fa* (month)	22	8.03	23.25	8.41		
Hamd scores	24.77	5.56	26.45	7.13		

* fa means first onset of age; ld means length of duration;

Methods

It is well known that interaction effects means that the interpretation of the individual variables may be incomplete or misleading. If we encounter the multiple regression without considering the interaction effect, we can express the model as:

$$\text{Metabolite} = b0 + b1 * \text{group} + b2 * \text{site} + \varepsilon' \qquad (1)$$

In equation (1), the unobservable statistical error is always denoted by the error term, ε'. Hence, it also implies that the interaction effect may exist. In this study, we treated ε' as the adjusted metabolite. It is shown as:

$$\varepsilon' = \textit{adjusted}\ \text{metabolite} \qquad (2)$$

$$= \textit{Metabolite} - (b_0 + b_1 * \textit{group} + b_2 * \textit{site})$$

As equation (3) shows, we then use ε to investigate whether the interaction effect exists.

$$\varepsilon = \textit{adjusted metabolite} - b_3 * (\textit{group} : \textit{site}) \qquad (3)$$
